# Comparing vaccination coverage before and during COVID-19 pandemic in children under one year in the health district of commune V in Bamako, Mali

**DOI:** 10.1186/s12887-023-04416-0

**Published:** 2023-11-27

**Authors:** Mountaga Diallo, Ilo Dicko, Samou Dembélé, Brahima Konaté, Cheick Oumar Doumbia, Ibrahim Sanogo, Ousmane Boua Togola, Drissa Konaté, Moussa Sangaré, Hawa Thiam, Yaya Ibrahim Coulibaly, Sory Ibrahim Diawara, Yacouba Toloba, Adama Balla Coulibaly, Mahamadou Diakité, Seydou Doumbia

**Affiliations:** 1 University Clinical Research Center (UCRC), University of Sciences, Techniques and Technologies of Bamako (USTT-B), Bamako, Mali; 2 Direction Générale de la Santé et de l’Hygiène Publique, Bamako, Mali; 3 Neglected Tropical Diseases Research Unit, University of Sciences, Techniques and Technologies of Bamako (USTT-B), Bamako, Mali; 4 Centre de Santé de Référence de la Commune V du District Sanitaire de Bamako, Bamako, Mali; 5 Centre Hospitalier Universitaire du Point G, Bamako, Mali; 6 Direction Régionale de la Santé du District Sanitaire de Bamako, Bamako, Mali

**Keywords:** COVID-19, Pandemic, Vaccination coverage, Routine vaccination, Children, Mali

## Abstract

**Introduction:**

Although an essential frontline service in the prevention of child morbidity and mortality, there are indications that routine vaccinations have been disrupted during the COVID-19 pandemic. The present study aimed to compare vaccination coverage before COVID-19 in Mali in 2019 and during COVID-19 in 2020.

**Objective:**

To compare vaccination coverages before COVID-19 in Mali in 2019 and during COVID-19 in 2020.

**Design:**

Cross-sectional study.

**Setting and participants:**

We collected routine immunization data from 2019 to 2020 of children under one year in the health district of Commune V in Bamako which includes twelve community health centers (CSCom).

**Results:**

Considering all vaccines together, coverage in 2019 was higher than in 2020 (88.7% vs. 71,6%) (p < 10^− 3^, Fig. 1). In 2020, low proportions of children vaccinated were observed in May (51.1%) two months after the first COVID-19 case in Mali on March 25, 2020. For all vaccines, the mean number of children vaccinated was significantly higher in 2019 (before COVID-19) as compared to 2020 (during COVID-19) (p < 0.05). However, in September and October 2019 BCG vaccine coverage was lower in 2019 as compared to 2020 (p < 10^− 3^).

**Conclusion:**

COVID-19 pandemic has affected routine childhood vaccine coverage in Commune V of Bamako, particularly in May 2020. Therefore, new strategies are needed to improve vaccine coverage in young children below 1.

## Background

Coronavirus disease 2019 (COVID-19) is an acute respiratory syndrome caused by the novel coronavirus SARS-CoV-2 transmitted from person to person through respiratory droplets and contaminated surfaces [1]. On March 2020, the World Health Organization (WHO) declared COVID-19 a pandemic and a threat to public health and health systems all around the world [2]. In October 2023, about 676 million COVID-19 cases were confirmed in the world [3] among which 9.5 million occurred in Africa [4]. The disease caused approximately 6.9 million deaths worldwide including 175,000 deaths in Africa [4]. In Mali, the first COVID-19 case was declared on March 25th, 2020 [5] and the country recorded about 33,000 confirmed cases and 743 deaths at the date of October 3rd, 2023 [3, 4]. This pandemic forced countries to take unprecedented preventive measures such as confinement, physical distancing, movements restriction, wearing masks, hands cleaning and public places closure [6]. These measures and public fear about the disease led to a drop in health services attendance [7, 8].

Like previous Ebola virus disease epidemics in some countries [9–11], COVID-19 disrupted smooth functioning of routine health services all around the world [12]. Approximately, 90% of world countries reported disruptions in health services’ attendance [12] with a particular impact on childhood immunization programs [13–16]. According to WHO, vaccine-preventable diseases threaten at least 80 million children under one year due to COVID-19 [17].

Malian Expanded Program of Immunization (EPI) launched in December 1986 targeted tuberculosis, measles, diphtheria, tetanus, whooping cough and poliomyelitis. Later, hepatitis B, meningitis A, yellow fever, *Haemophilus influenzae* type b, rotavirus, and *Streptococcus pneumoniae* infections were added (Table 1). Today, the EPI targets children from 0 to 24 months and makes possible the control of several childhood diseases by reducing mortality and morbidity related to vaccine-preventable diseases [18]. However, COVID-19 pandemic greatly affected Malian health services attendance [19–21]. According to previous studies, the number of routine medical visits dropped by 17% from March to July 2020. Pentavalent vaccination was reduced by 17.4%, BCG by 12% and Measles by 16.4% [20, 21]. Our study aimed to assess the change in routine immunization coverage before and during COVID-19 pandemic in the second most affected health district of Bamako in Mali.

**Table 1:** The immunization schedule for children aged 0 to 23 months in Mali

Age		Vaccines
**At birth**		BCG, OPV 0
**At 6 weeks**		OPV 1, Penta 1, PCV-13 1, Rota 1
**At 10 weeks**		OPV 2, Penta 2, PCV-13 2, Rota 2
**At 14 weeks**		OPV 3, VPI, Penta 3, PCV-13 3, Rota 3
**9 to 11 months**		VAR 1, VAA, MenAfrivac
**15 to 18 months**		VAR 2

Conducting this study will help to better understand the effect of the pandemic on routine vaccination program to serve as basis in tackling reemergence of vaccine preventable diseases when other epidemics arise.

## Methods

This was a cross-sectional study with retrospective data collection conducted in July 2021 in the health district of Commune V in Bamako. It covered vaccination data from March to December in 2019 and 2020. Malian health system is divided into three administrative levels: national, regional, and operational (health district levels). The national level is the system’s highest administrative level. The system is organized, directed, and recommended by the national level to the regional levels. Several health districts are under the jurisdiction of the regional level. A health district is made of a reference health center (CSRef) and a different number of dependent community health centers (CSCom) which offer a range of therapeutic, preventative and promotional health services. The health district of Commune V is constituted of the CSRef of Commune V and twelve CSCom.

Children under one year were the study population. Sampling was exhaustive of all routine immunization data from 2019 to 2020. Except one CSCom with incomplete data, all the CSCom of the health district of Commune V were included in our study. Vaccines of interest and their targeted diseases are summarized in Table 2.

**Table 2:** List of EPI vaccines and their targeted diseases

Vaccines	Diseases targets
**BCG** (Bacille Calmette-Guérin)	Tuberculosis
**VPO** (Oral polio vaccine)	Poliomyelitis
**VPI (**Inactived polio vaccine)	Poliomyelitis
**Penta** (Pentavalent vaccine)	Diphtheria, Tetanus, acellular Pertussis, Hepatitis B, *Haemophilus influenzae* type b
**PCV** (Pneumococcal conjugate vaccine)	*Streptococcus pneumoniae* diseases
**Rota** (Rotavirus vaccine)	Rotavirus gastroenteritis
**VAA** (Yellow fever vaccine)	Yellow fever
**VAR** (Measles vaccine)	Measles
**MenAfriVac** (Meningococcal A Africa vaccine)	Meningococcal meningitis A

### Data collection, management, and analysis

Sociodemographic data such as age and gender as well as vaccination-related data such as vaccination date, name of vaccines, number of children vaccinated, and number targeted were extracted from CSCom’s monthly activity reports and entered in Kobo-collect using smartphones. They were then downloaded as an MS Excel file before being exported into SPSS software (*Statistical Package for Social Sciences*) version 25 for further management and analysis.

Vaccination coverage **(**VC) was calculated using the formula VC = $\frac{t}{T}\times 100$ where *t* is the monthly number of children vaccinated and *T*, the expected monthly number of children to be vaccinated. The expected monthly number of children to be vaccinated in 2019 was T = 3,407, but in 2020 this number was not updated. We therefore deducted the expected monthly number of children to be vaccinated in 2020 from that of 2019, considering the annual EPI cohort growth rate of 4% (3,407+3,407 $\times$ 4/100 = 3,543.28). For multi-dose vaccines such as penta, rota, PCV, OPV, we calculated the average number of cumulative doses administered every month. For instance, the number of penta vaccine doses administered in July is the average number of doses administered for penta 1, penta 2 and penta 3 in July and so on and so forth. The decrease in coverage (%) was calculated using the formula $\frac{\left(n{\prime }-n\right)}{n}\times100$, where *n* is the number of children vaccinated in 2019 and *n’*, the number of children vaccinated in 2020. Student’s t test was used to compare the mean numbers of children vaccinated in 2019 and 2020. Vaccination coverage in 2019 and 2020 were compared using Pearson’s Chi-square test. The statistical significance level was set at 5%.

### Ethical aspects

Before the study starts, we obtained an administrative authorization from Bamako Health authorities, the Chief of Commune V health district, and Technical Directors of CSCom. Study data are stored in locked cabinets with limited access to authorized personnel.

## Results

**Fig. 1:**
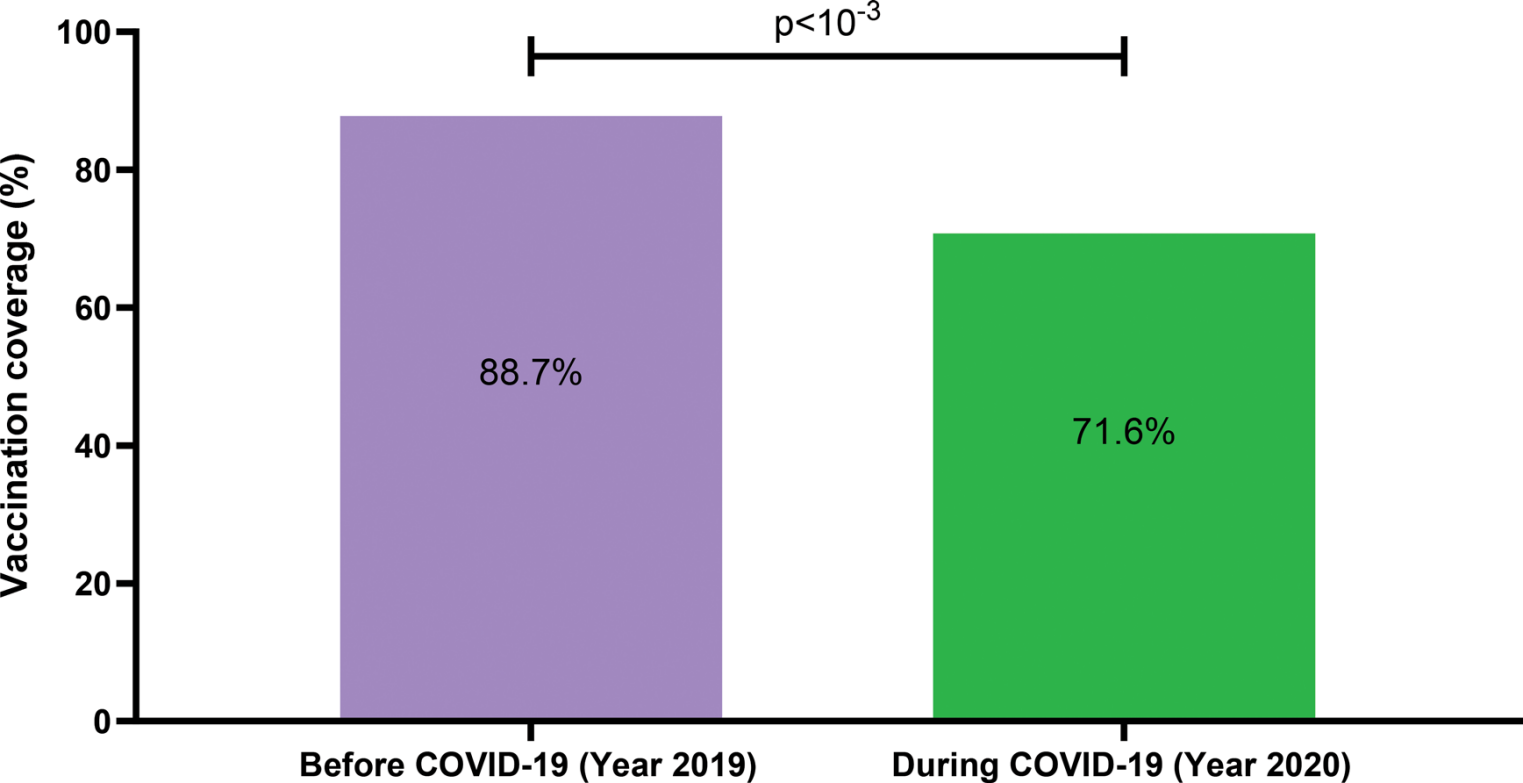
Variation in EPI vaccines’coverage before and during COVID-19.

**Fig. 2:**
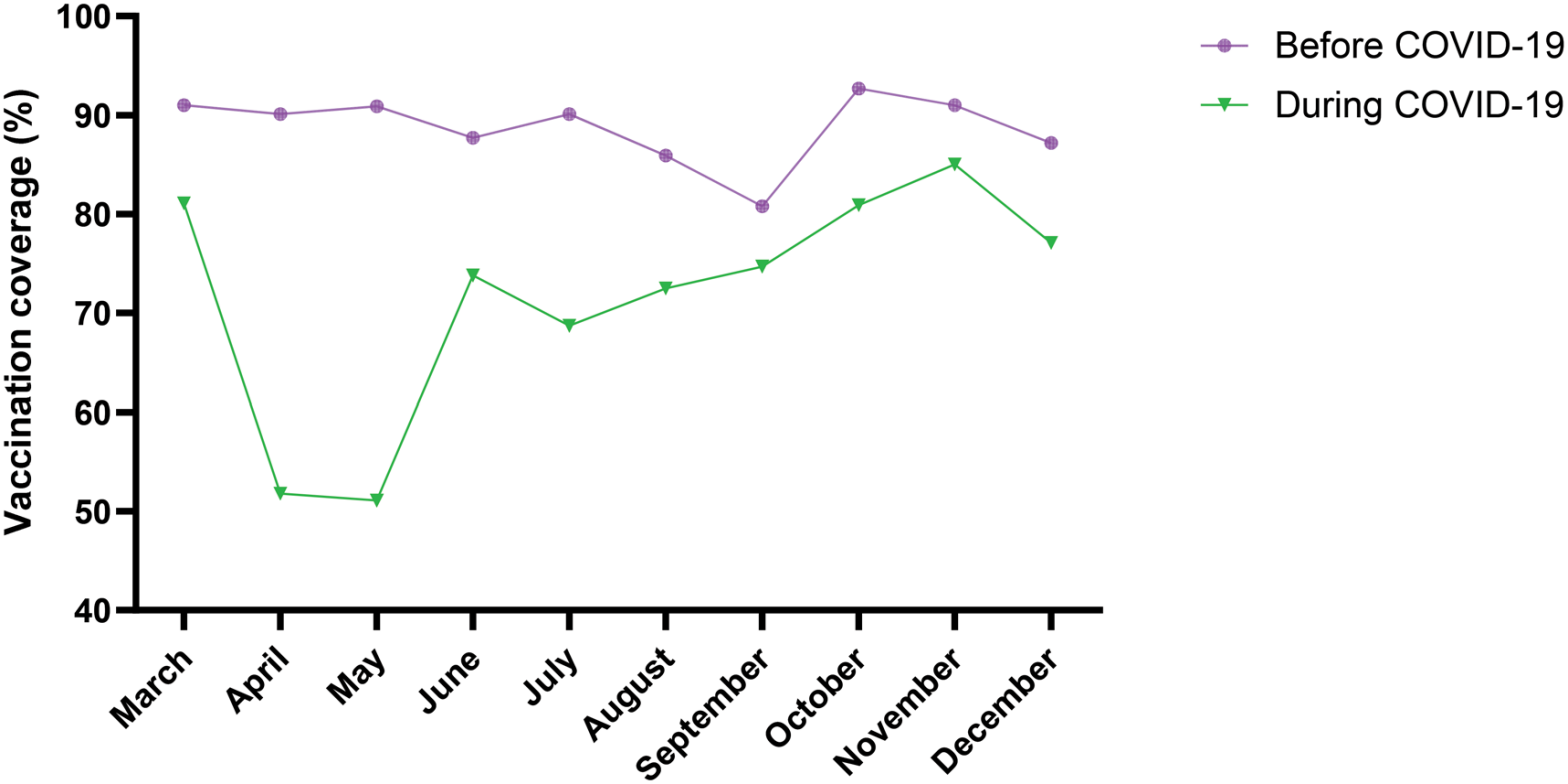
Variation in EPI vaccines monthly coverage before and during COVID-19

**Fig. 3:**
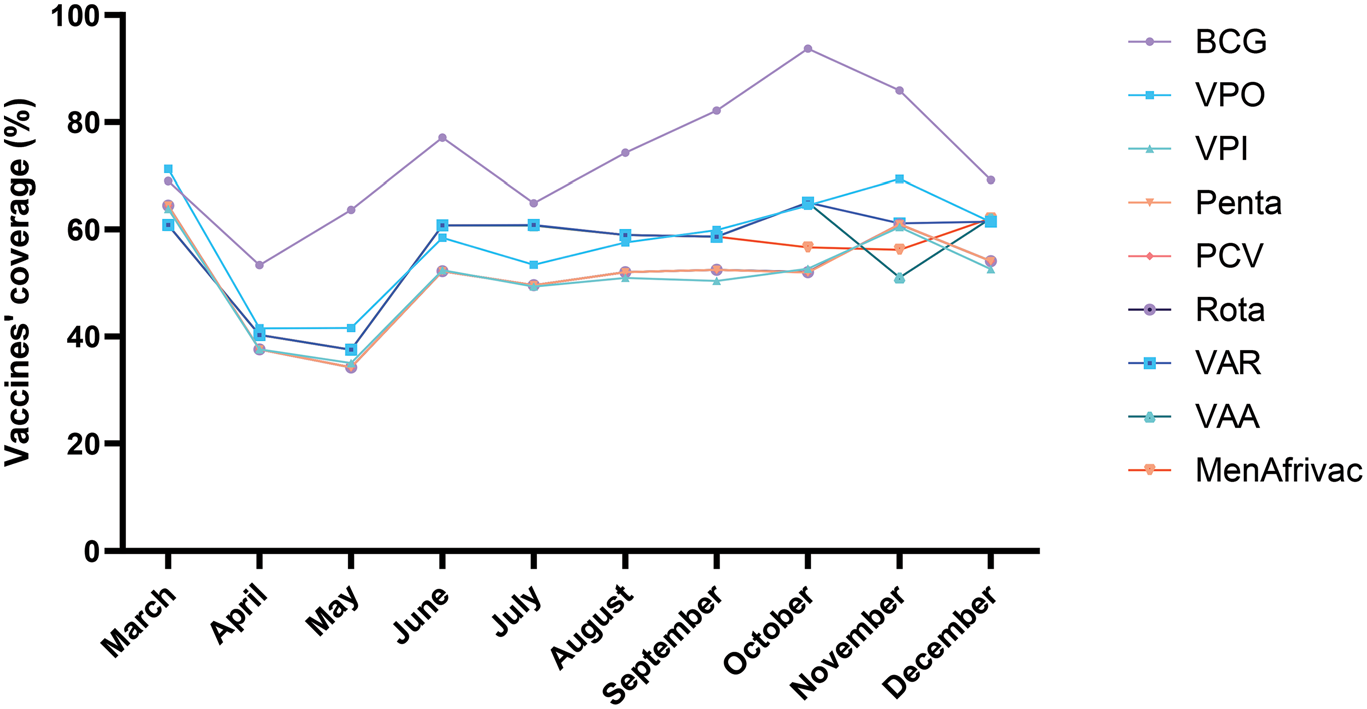
Monthly variation in vaccines’coverage during COVID-19

A total number of 31,346 children were vaccinated in 2019 as compared to 28,476 in 2020. More male than female children were vaccinated in 2019 while in 2020 it was the opposite with sex ratios of 1.12 and 0.97, respectively (Table 3). Considering all vaccines together, coverage in 2019 was higher than in 2020 (88.7% vs. 71.6%) (p < 10^− 3^, Fig. 1). In 2020, the lowest coverage was observed in May (51.1%, Fig. 2). For all vaccines, the mean number of children vaccinated was significantly higher in 2019 (before COVID-19) as compared to 2020 (during COVID-19) (p < 10^− 3^, Table 4) except BCG vaccine (Fig. 3). In September and October, BCG vaccine coverage was lower in 2019 as compared to 2020 (p < 10^− 3^, Table 5).

**Table 3:** Proportion of children vaccinated before in 2019 and during COVID-19 in 2020 according to sex

	Before COVID-19 n (%)	During COVID-19 n (%)
Male	16,557 (52.8)	14,049 (49.3)
Female	14,789 (47.2)	14,427 (50.7)
**Total**	**31,346 (100)**	**28,476 (100)**
**Sex ratio**	**1.12**	**0.97**

**Table 4:** Variation in mean number of vaccinated children by vaccine type before and during COVID-19 in health district of Commune V, Bamako

Vaccines	Before COVID-19	During COVID-19	p-value	Decrease (%)
BCG	2,932	2,598	0.037	(-11.4)
VPO	2,362	2,071	0.024	(-12.3)
VPI	2,216	1,798	0.002	(-18.9)
Penta	2,249	1,815	0.001	(-19.3)
PCV	2,227	1,817	0.002	(-18.4)
Rota	2,363	1,851	0.001	(-21.7)
VAR	2,207	1,894	0.011	(-14.2)
VAA	2,213	1,872	0.007	(-15.4)
MenA	2,217	1,863	0.003	(-16.0)

**Table 5:** Decrease in children vaccination in 2020 compared to 2019 according to vaccine type

Month	BCG	VPO	VPI	Penta	PCV	Rota	VAR	VAA	MenAfriVac
March	-16,5	3,2	2,6	-0,1	-0,1	11,6	-13,3	-13,3	-13,3
**April**	**-26,6**	**-40,5**	**-34,9**	**-45,3**	**-45,3**	**-46,0**	**-36,9**	**-36,6**	**-36,6**
**May**	**-33,0**	**-41,4**	**-43,0**	**-45,5**	**-45,6**	**-46,1**	**-39,8**	**-38,5**	**-39,6**
June	-9,7	-11,7	-5,9	-12,8	-12,8	-12,8	-15,2	-14,7	-14,7
July	-28,3	-22,0	-21,1	-21,0	-21,0	-21,0	-10,6	-10,7	-10,7
August	-14,4	-12,6	-31,7	-11,7	-11,8	-11,8	-10,1	-10,1	-10,1
September	13,3	16,1	-8,8	-7,1	-4,5	-7,1	-14,4	-14,4	-14,4
October	10,5	-7,0	-21,4	-23,9	-23,9	-23,8	-1,3	-1,3	-13,9
November	0,0	1,1	-8,0	-6,8	-6,7	-40,8	-7,2	-22,6	-14,7
December	-9,5	-7,9	-16,8	-19,0	-12,9	-19,0	7,2	8,3	8,3
**Total**	**-11,4**	**-12,3**	**-18,9**	**-19,3**	**-18,4**	**-21,7**	**-14,2**	**-15,4**	**-16,0**

## Discussion

Routine vaccination remains the cornerstone of public health practice in reducing morbidity and mortality in children due to vaccine-preventive diseases. In Commune V health district of Bamako, the capital city of Mali, vaccination coverage dropped by nearly 17.1% in 2020 compared to 2019. This could be explained by the parents fear for exposing their children to the risk of SARS-CoV-2 infection by attending CSCom as reported in Saudi Arabia [16, 22], in Italy [23], in Iran [24], in United States of America [25], and in Pakistan [26]. Other explanation such as unavailability of health personnel, constraints related to COVID-19 prevention measures and vaccine supply difficulties due to border closures and/or travel restrictions have been reported [16, 25, 27–29]. In 2020, a survey conducted by IMPRINT (Immunizing Pregnant Women and Infants Network) found that more than 50% of countries reported disruptions in delivery of maternal or infant vaccines [30]. Drop in vaccination coverage reflects the mistrust of vaccines that has been increasing worldwide for years, notably due to their composition, side effects and possible rumors of their links with certain diseases such as sclerosis, autism or cancers, or the fact that they are deemed useless or dangerous [31–34]. However, COVID-19 pandemic has accentuated this mistrust in scientific research, leading to a drop in vaccination coverage [35]. Previous studies have shown a spread of disinformation and rumors around COVID-19 leading to an increase in vaccination hesitancy during the pandemic [36–38].

In our study, a substantial drop in vaccine coverage has been observed in May 2020 compared to the same period in 2019. Since the first COVID-19 case on March 25th 2020 in Mali, attendance of child vaccination centers has declined in two subsequent months. However, from July 2020, vaccination coverage gradually increased to reach its peak in November 2020 (85.0%). Similar trends in vaccine coverage have been observed in United States of America [13, 39], in Italy [40], in Israel [41], and in Cameroon [42]. This increase may be related to decrease of COVID-19 fear after the first two months. As few people experienced severe COVID-19 or died from it, the population started to visit more frequently health care facilities. Similarly, adoption of preventive measures such as vaccination, hand washing, and physical distancing has contributed to improve attendance of health facilities and children vaccination center.

From our study, we have noticed that BCG vaccine coverage was less affected compared to other EPI vaccines. Indeed, BCG vaccine is administered to children at birth and in Bamako, the capital city, almost all the deliveries occur in health centers where BCG vaccine is made available for free to newborns. Similar findings on BCG vaccination have been reported in United States of America [11], in Saudi Arabia [16], in Japan [43], and in Cameroon [42].

Despite the overall decrease in vaccination coverage in 2020, an increase of vaccination coverage was observed with VAA, VAR, MenAfriVac in December, and with BCG in September and October as compared to 2019. This could be explained by a vaccine stock shortage experienced with these vaccines in 2019 in Bamako.

## Study limitations

Our study was conducted in one health district which is not representative of the capital city of Bamako. Therefore, the interpretation of these results could not be generalized to the entire population of Bamako. Using routine immunization coverage data from monthly reports produced by CSCom does not provide accurate information as observed with community surveys using information recorded on children’s immunization cards.

## Conclusion

COVID-19 has affected childhood vaccine coverage in Commune V of Bamako in Mali, particularly in May 2020. New strategies are needed to improve vaccine coverage particularly for young children in Mali.

## Data Availability

All data generated or analysed during this study are included in this published article.
